# Emission of Industrial Air Pollution and Mortality Due to Respiratory Diseases: A Birth Cohort Study in Poland

**DOI:** 10.3390/ijerph20021309

**Published:** 2023-01-11

**Authors:** Agnieszka Genowska, Birute Strukcinskiene, Jacek Jamiołkowski, Paweł Abramowicz, Jerzy Konstantynowicz

**Affiliations:** 1 Department of Public Health, Medical University of Bialystok, 15-295 Bialystok, Poland; 2 Faculty of Health Sciences, Klaipeda University, LT-92294 Klaipeda, Lithuania; 3 Department of Population Medicine and Lifestyle Diseases Prevention, Medical University of Bialystok, 15-269 Bialystok, Poland; 4 Department of Pediatrics, Rheumatology, Immunology and Metabolic Bone Diseases, Medical University of Bialystok, University Children′s Clinical Hospital, 15-274 Bialystok, Poland

**Keywords:** birth cohort, mortality, respiratory diseases, industrial air pollution

## Abstract

Background: Air pollution is a major risk factor for public health worldwide, but evidence linking this environmental problem with the mortality of children in Central Europe is limited. Objective: To investigate the relationship between air pollution due to the emission of industry-related particulate matter and mortality due to respiratory diseases under one year of age. Methods: A retrospective birth cohort analysis of the dataset including 2,277,585 children from all Polish counties was conducted, and the dataset was matched with 248 deaths from respiratory diseases under one year of age. Time to death during the first 365 days of life was used as a dependent variable. Harmful emission was described as total particle pollution (TPP) from industries. The survival analysis was performed using the Cox proportional hazards model for the emission of TPP at the place of residence of the mother and child, adjusted individual characteristics, demographic factors, and socioeconomic status related to the contextual level. Results: Infants born in areas with extremely high emission of TPP had a significantly higher risk of mortality due to respiratory diseases: hazard ratio (HR) = 1.781 [95% confidence interval (CI): 1.175, 2.697], *p* = 0.006, compared with those born in areas with the lowest emission levels. This effect was persistent when significant factors were adjusted at individual and contextual levels (HR = 1.959 [95% CI: 1.058, 3.628], *p* = 0.032). The increased risk of mortality was marked between the 50th and 150th days of life, coinciding with the highest exposure to TPP. Conclusions: The emission of TPP from industries is associated with mortality due to respiratory diseases under one year of age. A considerable proportion of children’s deaths could be prevented in Poland, especially in urban areas, if air pollution due to the emission of particle pollution is reduced.

## 1. Introduction

Mortality reported in children under one year of age is regarded as an important indicator of the health status of the population and may also serve as a reliable marker for the harmful effects of environmental pollution [1,2]. Thus, exposure to highly polluting materials, causing environmental pollution, is a significant factor that may affect the fetus and deteriorate processes during the prenatal period. These agents may lead to genetic alterations and deficits in fetal development as the developmental processes are strongly dependent on intrinsic maternal factors, which, in turn, may interact with environmental agents due to maternal exposure [3]. The inhalation of particulate matter (PM) causing air pollution during pregnancy may lead to oxidative stress and prolonged inflammation affecting embryonic development [4]. Subsequent deteriorations in the mother–fetus exchange have been shown to contribute to intrauterine growth restriction [5], low birth weight [6], and preterm delivery [7]. Intrauterine exposure to air pollution has an impact on birth weight and may confer a risk of pulmonary dysfunction in the fetus. Immaturity of fetal lungs, reflected by a reduced surface area for gas exchange and also hypoxemic mechanisms, is associated with various infections of the respiratory tract and an increased risk of mortality under one year of age. These data have been supported by a large body of evidence, based on a number of observational and case-crossover studies [8,9,10,11,12,13,14,15,16,17,18,19,20,21]. In addition to air pollution, several adverse birth outcomes may be caused by other agents and factors linked with oxidative stress and chronic subclinical inflammation, including nutritional deficiencies, smoking, and low socioeconomic status (SES) [4,22]. Parental profession, work conditions, and occupational status are also related to adverse birth outcomes [23,24]. 

According to a survey published by the European Environment Agency, Poland is one of the most polluted countries in Europe [25]. The major pollutants identified in the survey were benzo[a]pyrene compounds, PM, for instance, PM_10_ and PM_2.5_, and some toxic metals, including arsenic. It has been reported that the annual normative limits laid down by the European Union (EU) regulations were exceeded in many areas in Poland [25,26]. Although the emission of harmful agents and particles has considerably decreased since the 1980s due to the systemic overhaul and rapid restructuring of industries, industrial emissions continue to be responsible for air pollution in Poland in contrast to the vast majority of the EU [25,27]. According to our previous study, low air quality may explain, at least partly, the adverse birth outcomes at the population level, while mortality under one year of age may be associated with the pollution of air, water, and soil by industrial emissions [28]. In Poland, only one study on the association between environmental status and infant mortality, providing evidence of higher infant mortality in an area devastated by excessive industrial activity, has been published so far [29]. Some other studies have focused on the association between environmental exposure during the prenatal period and adverse birth outcomes [30,31].

Although the level of air pollution resulting from industrial activity in Poland is permanently high, the relevant data are still missing or incomplete, and there is a rationale to assess the association between environmental pollution and mortality due to respiratory diseases under one year of age. In the present study, we hypothesize that the differences in the rate of mortality due to respiratory diseases under one year of age are associated with the industries causing air pollution.

## 2. Materials and Methods

In this study, we analyzed a retrospective birth cohort of all live births in Poland, for individual cases under one year of age, which were reported from 2012 to 2017. The records used covered every live birth and death registered based on death certificates, as each death has to be mandatorily registered in Poland. The study included information about live births (*n* = 2,277,586) and deaths from these records (*n* = 9449). The extracted data were anonymous and contained additional information about live births (date of birth, sex of the child, birth weight, duration of pregnancy, age of the mother, and place of residence—defined at the administration level of 380 counties in Poland and identical with the parental residence) and deaths (the same as the dataset of live births and, in addition, the date and the primary cause of death).

To combine and link the anonymous datasets of live births and deaths, the information on the county of residence, date of birth, sex of the child, birth weight, duration of pregnancy, and age of the mother was matched. These variables and the procedures used were sufficient to link most of the birth–death cases, which resulted in a very high proportion of matching records (99.76%).

The aggregated dataset included the abovementioned variables and the number of children born adhering to the criteria and the number of individuals who died. The final dataset contained information on 2,277,585 children and a total of 9427 deaths (out of 9449), which was matched during the coupling procedure. At the end of the procedure, 22 cases remained unclear due to the disagreement between the datasets of births and deaths, which was possibly a result of potential errors, registry imprecision, or lack of adequate information, and these cases were removed from further analyses. Due to the lack of data for some covariates (birth weight—1418, duration of pregnancy—1666), a total of 1796 incomplete cases were excluded.

The time to death during the first 365 days of life was used as a dependent variable. We extracted, specifically, 248 deaths due to respiratory diseases under one year of age (Table 1), as defined by the codes J00–J99 of the International Statistical Classification of Diseases and Related Health Problems, Tenth Revision, ICD-10 by the World Health Organization (WHO) [32]. In the survival analysis, the observation time from the birth date was calculated in days (as no precise data on the exact hour of birth and decease were available) as the difference between the birth date and the date of death. For children (newborns and infants) who survived the whole observation period (1 year), the observation time was set as 365 days, whereas for the cases with birth and death on the same day, an appropriate correction was implemented to avoid bias in results (0.25 days instead of 0).

### 2.1. Assessment of Environmental Factors

Harmful emission of air pollution was described as total particle pollution (TPP) and was used as a marker of industrial activity [33]. The TPP is an emission of total dust particles from factories and industrial plants (fuel combustion, cement/lime, silicon, chemical fertilizers, surfactants—surface-active agents and polymers, carbon—graphite, carbon black, coal, and polymers, chromium, mercury, lead, cadmium, arsenic, zinc, manganese, polycyclic aromatic hydrocarbons). Air emission of TPP from industries was estimated using the official data derived from the mandatory reports on the emission of air pollution (OS-1) published by the Central Statistical Office (CSO) in Poland [34]. Data on TPP concentration in ambient air were site-specific, i.e., obtained with regard to territorial division using administrative units, called TERYT (National Official Register of the Territorial Division of the Country) [35]. To investigate the relationship between air pollution and matched birth with death occurring under one year old in Poland we used 380 counties. Counties are relatively small units to represent environmental factors, and the requirement for sufficiently sized units permits robust analyses of any association between air pollution and child health status. The mean annual number of live births recorded per county was 1002 (range 177–19,824). The mean area of the county was on average 826 km^2^ and ranged from 13 km^2^ to 2975 km^2^.

We predefined the assessment of the exposure as the estimation of the emission rate because the emission of TPP could presumably lead to, and be translated to, exposure to TPP. For each matched birth with death, exposure to TPP was estimated based on the annual measurements in tons per km^2^ registered within the counties in which occurred industrial activity [36]. To calculate the exposure of the study population we used information on TPP in counties of parental residence as reported on the birth certificate. Between 2012 and 2017, there were no industries in 35 counties, and therefore, the level of TPP was estimated at 0 tons/year. This was related to the counties with a population density of 27–296 inhabitants per km^2^ and also to one urban resort county with 2156 inhabitants per km^2^. The total area of non-industrialized regions was 30,595 km^2^ (i.e., 9.78% of the total area of the country).

### 2.2. Assessment of Additional Covariates

Covariates considered for inclusion in the models that deal with the association between air pollution and mortality due to respiratory diseases encompassed the following individual characteristics of newborns and mothers: sex of the child (male, female), birth weight (low birth weight <2500 g, normal 2500–3999 g, high birth weight ≥4000 g), duration of pregnancy (preterm <37 weeks, term-born 37–41 weeks, post-term >41 weeks), and age of the mother (<20, 20–29, 30–39, and ≥40 years). In addition to the data derived from individual records, we took into account some potential demographic factors (urbanization) and SES (unemployment and housing area) related to the contextual level [37,38]. We controlled our analysis in terms of urbanization to better represent the emission of air pollution; this was performed by reducing the likelihood that any reduction in the risk of mortality due to the lower level of TPP would be incorrectly attributed to the less urbanized areas. Thus, we included the level of unemployment and housing conditions to adjust for SES, which can modify the effects of the concentration of air pollution on infant mortality [17].

Additionally, the complete information concerning the emission of air pollution, urbanization, unemployment, and housing area was assigned to a given year and county of birth, based on the territorial structure and a specific geographical county code TERYT. Information on live births and mortality, emission of air pollution, level of urbanization, unemployment, and housing area over the years 2012–2017 was obtained from the CSO. 

### 2.3. Statistical Analysis

The analysis of survival was performed using the Cox proportional hazards model. The assumption and appropriateness of the proportional hazards concept were ascertained by testing whether the scaled Schoenfeld residuals were independent of survival time. No significant violations in the proportional hazard assumption were found for either of the variables incorporated in the Cox model. To demonstrate the estimated hazard functions (h(t)), the eighth-degree polynomial interpolation of cumulative hazard functions h(t) was carried out, and the first derivative of these polynomials was used for the approximation of h(t). Some of the variables (i.e., emission of TPP, urbanization, unemployment, and housing area) were not assessed individually but analyzed in the context of a given geographical region. Therefore, while composing the statistical models, there was a necessity to consider the correlation of the above variables at the county level. We used robust estimates of the standard errors that took possible dependence between observations into account. In the statistical analyses, TPP was not treated as a continuous variable, but as a variable categorized into 5 subgroups based on quintile values, in order to avoid model mismatches in case of potential non-linearity or non-monotonicity of the relationship. 

Three models were constructed to check the assumed associations and verify the hypothesis. Model 1 included only TPP. Model 2 included TPP and individual covariates (sex of the child, birth weight, duration of pregnancy, and age of the mother) as these parameters were considered to be linked with birth outcomes. Model 3 included the contextual factors analyzed in Model 2 and also some demographic factors and SES, including urbanization, unemployment, and housing area. Results were presented as hazard ratios (HRs) with 95% confidence intervals (CIs).

The statistical analyses were conducted using software R version 3.6.1 with the Survival Analysis package version 2.44-1.1. The hypotheses were verified at a significance level of 0.05.

## 3. Results

There were 1,170,495 male live births and 1,107,090 female live births (51.4 vs. 48.6%) included in the analysis. Out of the 9427 cases of death incorporated in the dataset, 248 had occurred due to respiratory diseases under one year of age, of which 147 were male (59.3%), whereas 101 were female infants (40.7%). Among these respiratory-disease-related deaths, the vast majority were caused by pneumonia (ICD-10 classification: J12–J18), which indicates that 226 deaths were assigned (91.12%) (Table 1).

Children who died due to respiratory diseases had a lower birth weight, and their mothers had a shorter duration of pregnancy. In addition, their mothers were younger compared to those of the children who survived. The distribution of TPP emissions from industries was found to be higher in the counties where deaths had occurred; these areas were more urbanized and also demonstrated a higher level of unemployment (Table 2). 

The rate of mortality due to respiratory diseases was estimated at 10.89 per 100,000 live births and differed depending on the quintile for the emission level of TPP; the mortality rate was particularly high for the fifth quintile: 16.10 per 100,000 live births (Table 3).

Twenty percent of children born in the 58 counties with the highest level of emission (ranging from 1.23 to 22.47 tons/km^2^ in the fifth quintile for TPP) were the residents of those counties. The total area of the counties was 10,067 km^2^, and the number of residents in the counties was 10,464,251. The mean population density was 1536 inhabitants per km^2^ (91–3364), the proportion of the urban population was 93.55% (41.14–100.0%), the proportion of unemployed was 9.52% (2.87–19.5%), and the mean housing area per one person was 25.6 m^2^ (21.9–33.0 m^2^). Out of those 58 counties, the majority were urban—53 were cities, whereas the remaining counties were mixed urban–rural areas. Many of these 58 counties formed a continuous geographic area (conurbation of 13 cities), which was located in Southern Poland. This region is the most industrialized with several heavy industry sectors (mining, power generation, vehicle production, metallurgical, and chemical industries) operating at an area of 1802 km^2^ in a specific geographical pattern (adjacent counties with a high population density).

Table 4 displays the unadjusted and adjusted HR of the association between the increase in the emission of TPP and the rate of mortality due to respiratory diseases. From the results, we did not find any relationship between TPP and mortality due to respiratory diseases within quintiles 2, 3, and 4. In contrast, in the highest quintile, a significant association was found between an increase in TPP emissions from 1.23 to 22.47 tons/km^2^ and mortality due to respiratory diseases. In the unadjusted Model 1, which included only the level of TPP emission, the rate of mortality of children attributed to the fifth quintile of TPP emission was higher compared with the rate of mortality of children born in counties with the lowest levels of emission (HR = 1.781 [95% CI: 1.175, 2.697], *p* = 0.006). Model 2, which was adjusted for the individual characteristics of newborns and mothers, showed that the mortality rate of infants born in the regions at the fifth quintile of TPP emission was significantly higher than that of the children born in counties with the lowest levels of TPP emission (HR = 1.798 [95% CI: 1.205, 2.682], *p* = 0.004). After additional adjustment with the inclusion of demographic factors and SES (Model 3), the effect observed was almost twofold with a higher risk of mortality among the children exposed to the TPP emission in the fifth quintile compared to the mortality rate among the children born in counties with the lowest TPP emission (HR = 1.959 [95% CI: 1.058, 3.628], *p* = 0.032).

**Figure 1:**
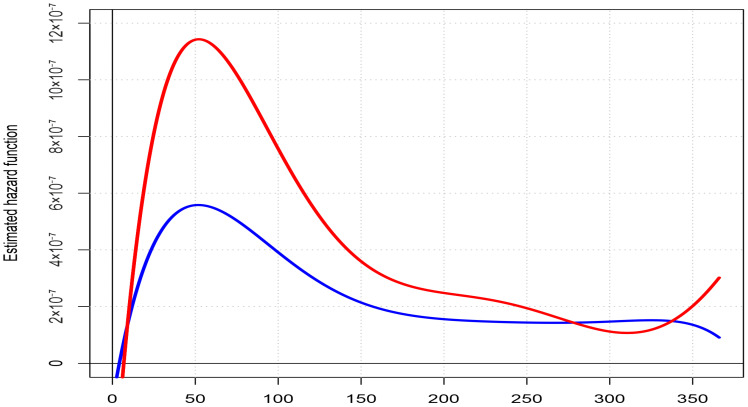
The estimated hazard curves for the deaths due to respiratory diseases in the population of live births delivered between 2012 and 2017 in counties in Poland with lower and higher emissions of total particle pollution. Legend: lower (first quintile)—blue line; higher (fifth quintile)—red line.

The estimated hazard curves are shown in Figure 1. The blue line denotes the risk of mortality of the children born in the areas with lower TPP emissions in the four lower quintiles, whereas the red line denotes the risk of mortality of the 20% of children born in the areas exposed to the highest TPP emissions (fifth quintile). An increase in death risk was observed within a similar period in both groups, which was higher for children born in the areas with higher TPP emissions. 

**Figure 2:**
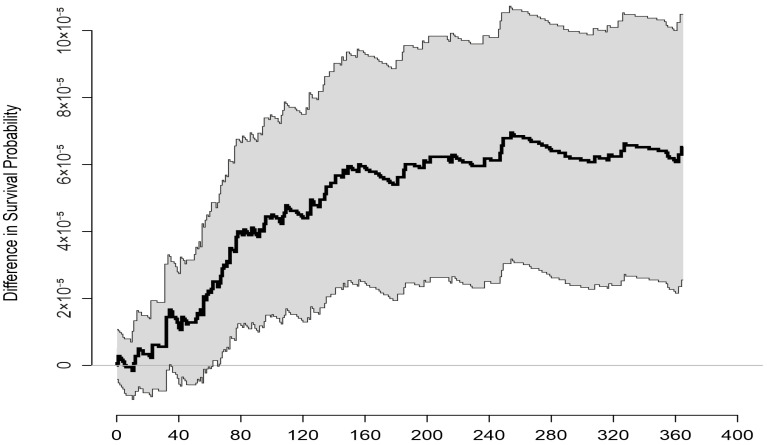
Differences in the survival probability for the population of live births from counties with the lower (first quintile) and higher (fifth quintile) levels of emissions of total particle pollution with 95% confidence interval (grey area).

Figure 2 demonstrates the differences in the survival rate among the children exposed to different levels of TPP emissions. The difference is particularly expressed and increases at approximately 50–150 days of age. Before and after this timeframe, the differences related to the exposure to TPP emissions are stable.

## 4. Discussion

In this study, the pattern of air pollution caused by the industrial emission of TPP was found to considerably vary across the country depending on the location and the area of residence and was assumed to have been responsible for the increase in mortality due to respiratory diseases under one year of age. A significant negative impact was found for respiratory diseases in infancy, as an association between mortality and the upper quintile of TPP emission was found. Importantly, after including the covariates such as neonatal and maternal characteristics and other factors (urbanization and SES), the estimated HR for TPP remained consistent and valid with that of the main analyses. The alarming figures provided evidence of a serious public health issue potentially resulting from the uncontrolled development of industries in Poland.

Our results are largely consistent with other studies indicating a relationship between particle pollution and mortality due to respiratory diseases under one year of age [8,9,10,11,12,13,14,15,16,17,18,19,20,21], although some less-representative studies did not find any such association [39]. In a report from the Czech Republic—an industrialized neighborhood country—the same pattern of mortality due to respiratory diseases in relation to the emission of total suspended particulates was found, particularly in the fifth quintile [8]. By analogy, no significant associations between mortality in infants and the emission of total suspended particulates at the four lower quintiles were found in the present study, but the highest quintile of the emission of total suspended particulates coincided with mortality, even after adjustment for additional variables. On the other hand, in a survey conducted on the Mexican population, low or intermediate SES and poor living conditions were identified as important confounders that leveled out the link between air pollution (PM_10_) and infant mortality [16]. Thus, the multivariate or multifactorial nature of mortality is of value and should be included in the analyses to prevent misinterpretation. In the present study, these covariates were controlled: the characteristics of newborns and mothers at the individual level and SES at the contextual level. Therefore, the inclusion of these covariates with a potential impact on mortality in the model did not eliminate the independent impact of TPP.

Our study showed a specific increase in the risk of mortality due to respiratory diseases between the 50th and 150th days of life. This might have been possible due to a cumulative effect of critical negative factors during early infancy, for example, immunological immaturity and deteriorated response to infections, poor defense mechanisms, incomplete vaccination schedule, or transient iron deficiency [40,41,42,43]. There is also a possibility that some additive physiological or pathogenic mechanisms are involved in exacerbating the health issues, including an increased risk of morbidity and mortality between the second and the fourth month of age. These considerations, however, do not detract from the main findings of our study.

Newborns and infants are particularly vulnerable to air pollution of industrial origin [44]. According to some recently published studies, ambient pollution may considerably contribute to the increased proportion of hospital admissions for respiratory diseases in children. In particular, a high concentration of PM_10_ the day before hospitalization has been recognized as most responsible for a longer duration of hospitalization of children and for the economic burden of treatment due to respiratory diseases in this population [45]. It has been reported that susceptibility to death due to exposure to air pollution is greater in neonates (immediately after birth) and infants compared with adults: 14.2% vs. 2.3% [11]. According to a report from Japan, negative birth outcomes were associated with concentrations of PM below the standardized lower limit causing air pollution [17]. A recent longitudinal study, performed in the United Kingdom on a population-based birth cohort, demonstrated that intrauterine and early life exposures to air pollution (PM_10_) may consequently produce a significant, though small, negative impact on lung function at the pre-pubertal stage [46]. There is still insufficient evidence available in this field due to the limited number of published data and studies. Therefore, research evaluating the effects of excess exposure to industrial pollutants on the health status of the youngest age group has become a priority in the WHO European region [33]. Noticeably, the mortality associated with respiratory diseases, in particular pneumonias, throughout the life course can be potentially reduced by medical intervention. Furthermore, a large number of live births in Poland (approximately 380,000 per year) may affect not only the health of neonates and infants but also the demographic structure and health outcomes of the population in the future. There is evidence that antenatal exposure of a fetus to air pollution, particularly during lung development, may lead to respiratory diseases both in childhood (e.g., asthma or allergy) and in adulthood (e.g., chronic obstructive pulmonary disease), although the causal pathway remains to be explained with caution [47,48]. Interestingly, some studies indicate that exposure to local environmental pollution in early life stages may even play a role in shaping human capital and, what is more, economic outcomes in the future. Exposure to air pollution during the prenatal period may further minimize opportunities to achieve higher mobility in the job market by children from families of low socioeconomic status [49]. In this view, industrialization leading to the deterioration of the health status of adults, including women of reproductive age, escalates the subsequent environmental inequalities in health. Hence, there is unquestionably a need to implement well-designed strategies and undertake systematic activities aimed at reducing air pollution in Poland. This may be a significant, alarming appeal addressed to governmental agencies, policymakers, and health authorities, particularly in the era of progressing climatic changes worldwide.

Our study used a very large database for investigating the association between mortality due to respiratory diseases in children and the emission of total particle pollution attributed to all branches of industry in Poland. Importantly, this analysis focused only on industrial sources of pollution, so there was no confounding effect of other origins of pollution such as furnaces or road transport. The six-year-long observation period of our study reassured the reliability of the data and the stability of the pattern of TPP emissions encompassing the entire area of the country, so the collection of potentially biased or selective data, for example, a study population chosen exclusively in terms of the proximity of pollution, was avoided. Most importantly, in the Central and Eastern European regions, only one historical study has been published so far, authored by Czech investigators [8,10]. 

We obtained access to a dataset of over two billion live births, thereby reassuring a high statistical power for the complete analysis of rare demographic events, including 248 death cases due to respiratory diseases, accounting for 2.64% of the total figure of deaths under one year of age. The number of variables used in this study was limited; nevertheless, the most significant factors associated with the mortality of children were included, from the available birth and death certificates. The linked variables used in the analysis (the sex of the child, birth weight, the duration of pregnancy, and the age of the mother) allowed an accurate matching of births and deaths up to 99.79%, indicating a high quality of data collection, e.g., 86% [10] and 94.3% [15]. The study results revealed the situation associated with the environmental inequalities in the health sector in Poland and offered insight into understanding the geographical pattern and background of the inequalities in the health of neonates and infants. The identification of the geographical regions with an increased risk of mortality in children less than one year of age is valuable and may certainly be useful in designing local or national health policies. The original representative data, obtained from one of the largest databases in Europe, and the completeness of the results warrant further possible research.

In this study, we used quintiles for the analysis concerning the emission of industrial air pollution, and mortality due to respiratory diseases. Our choice to treat exposure to air pollution in terms of quintile groups was motivated by doubts about the linearity of the nature of the relationship between exposure and health outcomes. Dividing into groups based on quantile results in groups of similar size maximizes the statistical efficiency of comparisons between them, although, of course, at the same time, causes the ranges of these intervals to have different widths, depending on the shape of the distribution. In particular, the highest quintile encompassing the tail of the distribution is wider, which, however, is a natural consequence. As for the choice of the number of subgroups we could, of course, have made a division based on the median or tertiles, but in our opinion, this would have given an insufficiently accurate assessment of the nature of the relationship. We considered the partition into five groups to be an optimal compromise: a sufficiently large number of categories for estimating the monotonicity and linearity of the studied relationship, while not making the data too fragmented.

The results of this study should be interpreted in light of some limitations, indeed. The data used in this work concerned the individual cases of live births and deaths due to respiratory diseases; however, few birth and death records were incomplete. Therefore, to avoid the confounder effect and minimize the influence of missing information, the records containing incomplete data were excluded. The proportion of live birth records lacking information about the individual characteristics of newborns and mothers was only 0.08%, and thus, the analyses may be regarded as reliable. We analyzed the mean values of TPP emission in the counties but did not assess the individual exposure using TPP concentrations obtained from the daily estimates generated by monitoring devices at the county level. We assumed that the exposure to TPP could have resulted from TPP emission and investigated the total emission in 380 counties; however, in Poland, the measurements had been performed in 226 settings for PM_10_ and 91 settings for PM_2.5_ [50]. Therefore, it was not feasible to obtain overall estimates from monitoring devices in all counties. The exposure to TPP was determined at the aggregated level given that the birthplace was derived from the TERYT coding system for a single county. We are aware that TPP measurements were based on annual industrial emissions in the counties. This may have led to certain misclassification errors regarding exposure or underestimated results because of the differences in the volume of atmospheric emission within the limits of a single county. Furthermore, we did not have access to information about smoking during pregnancy or changes in the place of residence (migration) either, but these variables would have a minor or trivial effect. Moreover, there are also other sources of PM causing air pollution beyond the TPP from industries, including burning fossil fuels, nonindustrial combustion processes, and road transport [51]. We believe that, despite the abovementioned limitations, our estimates are well grounded and consistent with several reports assessing the exposure to pollution using an approach based on address and county of residence [8,15,16,17]. 

It is uncertain whether our results may be generalizable throughout Europe or to all populations, as the industrial distribution and map of air pollution may considerably differ between countries. However, the data strongly support the association between the detrimental effect of TPP and respiratory-disease-specific mortality in children, indicating that this significant public health problem needs a reasonable solution, and it is necessary to rethink some of the ground rules of industry organization in Poland.

## 5. Conclusions

This study showed that the emission of TPP from industries in Poland is associated with mortality due to respiratory diseases under one year of age. The results suggest that both individual- and contextual-level factors modify this effect, but the finding does not detract from the main conclusion. Therefore, well-designed strategies should be urgently implemented at the national level to alleviate the detrimental effect of air pollution caused by industries on children’s health in the future.

## Figures and Tables

**Table 1:** The number of deaths under one year of age due to respiratory diseases according to the specific ICD-10 codes.

ICD–10	N (%)
J00–J06: acute upper respiratory infections	2 (0.81)
J09–J11: influenza	3 (1.21)
J12–J18: pneumonia	226 (91.12)
J20–J22: other acute lower respiratory infections	5 (2.02)
J40–J47: chronic lower respiratory diseases	1 (0.40)
J60–J70: lung diseases due to external agents	5 (2.02)
J80–J84: other respiratory diseases principally affecting the interstitial tissue	3 (1.21)
J95–J99: other diseases of the respiratory system	3 (1.21)
Total	248 (100.0)

**Table 2:** Characteristics of children who died due to respiratory diseases in relation to other children in Poland (average for the 2012–2017 period).

Variable Name (Unit)	Deaths under One Year of Age Due to Respiratory Diseases (*n* = 248)	Children Who Survived or Died Due to Other Causes (*n* = 2,277,337)
	Mean ± SD (Q1/Me/Q3)	Mean ± SD (Q1/Me/Q3)
Total particle pollution at the county of residence (tons/km^2^)	1.06 ± 2.54 (0.03/0.15/1.45)	0.73 ± 1.63 (0.02/0.11/0.99)
Birth weight (g)	2729.64 ± 811.14 (2200/2895/3307)	3357.57 ± 558.46 (3060/3400/3700)
Duration of pregnancy (weeks)	37.05 ± 3.73 (36/38/40)	38.91 ± 1.90 (38/39/40)
Age of the mother (years)	27.87 ± 6.38 (23/28/32)	29.09 ± 5.20 (26/29/33)
Urban population at the county of residence (%)	63.83 ± 30.98 (36.85/58.29/100)	59.07 ± 31.24 (33.27/53.66/100)
Unemployment at the county of residence (% of the population)	11.10 ± 6.43 (5.7/10.1/14.3)	10.98 ± 6.09 (6.1/10.2/14.7)
Housing area at the county of residence (m^2^ per inhabitant)	26.80 ± 3.08 (24.60/26.50/28.60)	26.94 ± 3.12 (24.8/26.5/28.6)

Values are presented as mean ± standard deviation (first quartile/median/third quartile).

**Table 3:** Mortality due to respiratory diseases under one year of age in quintile groups of TPP emission.

	TPP					Total
	Q1 0–0.2	Q2 0.03–0.06	Q3 0.07–0.24	Q4 0.25–1.22	Q5 1.23–22.47	
Rate of mortality * (number of cases)	9.04 (*n* = 52)	11.37 (*n* = 42)	8.65 (*n* = 37)	9.74 (*n* = 44)	16.10 (*n* = 73)	10.89 (*n* = 248)

TPP—total particle pollution (in tons/km^2^); Q—quintile. * Rate per 100,000 live births.

**Table 4:** Risk of mortality due to respiratory diseases under one year of age associated with the emission of TPP.

TPP (Tons/km^2^)	Model 1	Model 2	Model 3
		HR (95% CI)	
Q1 [0–0.2]	Ref.	Ref.	Ref.
Q2 [0.03–0.06]	1.258 (0.786, 2.014)	1.233 (0.770, 1.974)	1.220 (0.760, 1.957)
Q3 [0.07–0.24]	0.957 (0.586, 1.563)	0.939 (0.576, 1.531)	0.925 (0.541, 1.580)
Q4 [0.25–1.22]	1.078 (0.688, 1.688)	1.083 (0.691, 1.697)	1.143 (0.637, 2.050)
Q5 [1.23–22.47]	1.781 (1.175, 2.697) **	1.798 (1.205, 2.682) **	1.959 (1.058, 3.628) *

TPP—total particle pollution; HR—hazard ratio; CI—confidence interval; Q—quintile; and Ref—reference category. Model 1: unadjusted model with only TPP. Model 2: TPP adjusted for the characteristics of mothers and newborns. Model 3: TPP adjusted for the characteristics of mothers and newborns, demographic factors, and socioeconomic status. ** *p* ≤ 0.01; * *p* ≤ 0.05.

## Data Availability

Data were collected from public datasets analyzed or generated during the study and are presented in Table 1 and Table 2.
